# Artificial Teeth

**Published:** 1867-04

**Authors:** 


### ARTIFICIAL TEETH.

ANONYMOUS.

*Mr. President:*—Feeling the obligation resting upon me, as a member of this Association, to do something if it be but little, to sustain and make this meeting one of interest to us all, I thought best to profit by the prompting of our worthy Secretary to make a few suggestions in regard to the improvement of artificial teeth, feeling, however that the remarks which may be drawn out upon this subject from other members of this association, will be of much more interest than anything I may say, hoping, however, that our ideas and suggestions, as a whole, may add some little impetus to the ball of progress which has been moving forward so fast during the last fifteen or twenty years.

Who of us, as we look over the almost endless variety of shade, color, form, size and style of teeth adapted to all styles of work which we now have furnished to our hand, would have any desire to go back to the old carved blocks of ivory or the old painted gums, and first efforts of any of our manufacturers of artificial teeth? None of us, I think, who had experience in those days would like to be set back to the old starting point and lay aside the improvements of to-day. In looking back we see what has been accomplished in the past, and while we fully appreciate the advancement of the present time, may we not hope for a still greater advancement in the future, if we but *work earnestly* and labor on, each day witnessing some improvement which will be a step forward in the great work we have to accomplish.

The great object to be attained in arranging an artificial denture, should be to provide our patients with the very best substitute for the lost organs that it is possible for us to give them. Something that will be useful as well as ornamental, which will not only restore the features to their original form and beauty, but will enable them to masticate their food with comfort and ease. To accomplish both of these results is not always an easy task. For instance, you have a full upper denture to insert, the jaw is fully developed with thick, heavy alveolar border, the inferior jaw narrow and contracted, and perhaps the bicuspids bowed upon their outer face so as to throw the grinding surface of these teeth still further in. With continuous gum work there is not so much trouble in cases of this kind. You can arrange the teeth out or in to suit your pleasure, and lay on the gum accordingly; but with the rubber work it is more difficult. The gum of these teeth are almost on a plain with the face of the teeth, the upper edge of the gum curving slightly back. If you attempt to get a proper articulation with the under jaw, you have to set the points of them *in* very much, giving them a contracted appearance across the cutting edge, or else grind the gum almost entirely away, and setting the neck of the tooth further in. I have often felt the need of, and have tried to find teeth with the gum curved out from the face of the tooth more or less as the case may be, which would let the neck of the tooth drop in without cutting the gum all away, also a greater thickness of tooth in the bicuspids and molars through from the outer face to the lingual surface, giving them a good broad articulating surface. A tooth of the same character with the cutting edge of the incisors and cuspids slightly inclined inwards, is often needed for the under jaw when that is fully developed, and the upper contracted and narrow, so the articulating surface may be brought well in, and more security given to the plate in mastication.

There are some few general defects in artificial teeth I think, which could be easily remedied. The lower incisors are too narrow, and the cutting edges are not curved inward as much as I would like in order to get good articulation, and the lower bicuspids are not bowed sufficient upon their outer face, leaving their outer cusp too full and prominent. I also think that a good many of the bicuspids and molars, both above and below, lack articulating surface, not thick enough through from the buccal to the lingual surface, and in a good many cases, the superior lateral incisors are not proportionately large. I think if you will take pains to compare them with the natural teeth, you will find that to be the case. There are also defects in shape and color, which I will not attempt to point out at this time, which you have all no doubt experienced. My experience and judgment may not correspond with many others present, and, no doubt, others have discovered defects which I have failed to see, or may differ with me entirely in regard to those points which I have mentioned. We are here to compare notes, and suggest ideas, and to profit by each other's experience. Let us have the expression of every one present upon this subject, as there is room for improvement; and let us hope that the day is not far distant when the combined efforts of so many workers in the same direction may result in producing the "*ne plus ultra*" of all our hopes and expectations.

